# Dr. Arthur Burrows Rood

**Published:** 1914-12

**Authors:** 


**Dr. Arthur Burrows Rood,** Syracuse 1889, died at his home in Minoa, N. Y., of septicaemia, August 27, aged 50. He was an ophthalmologist.

